# Travelling through time with Disease Models & Mechanisms

**DOI:** 10.1242/dmm.052259

**Published:** 2025-02-24

**Authors:** Saanjbati Adhikari, Rachel Hackett

**Affiliations:** The Company of Biologists, 94 Station Road, Histon, Cambridge CB24 9LF, UK

## Abstract

**Summary:** This year, The Company of Biologists − publisher of Disease Models & Mechanisms (DMM) − is celebrating its 100-year anniversary. In 2025, we discuss DMM's journey since its launch in 2008.

**Figure:**
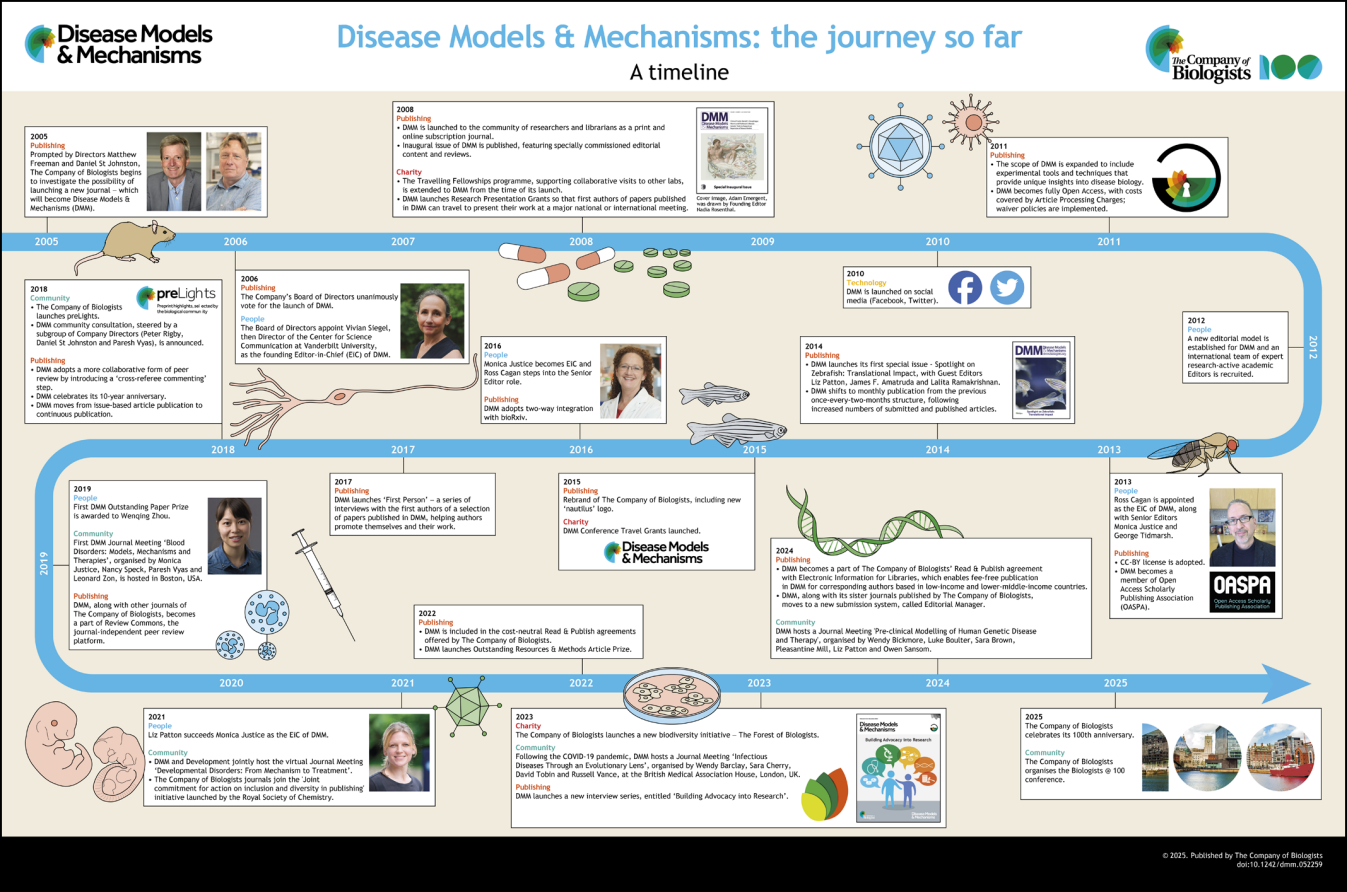
See supplementary information for a high-resolution version of the poster.

The Company of Biologists, the not-for-profit publisher run by research-active scientists, is celebrating its 100-year anniversary in 2025. As we commemorate this remarkable milestone, we aim to shine a spotlight on the evolution of the Company's five specialist journals: Development, Journal of Cell Science, Journal of Experimental Biology, Disease Models & Mechanisms and Biology Open.

Disease Models & Mechanisms (DMM) is an international peer-reviewed Open Access (OA) biomedical journal. DMM was founded in 2008 with the goal to “foster a connection between the model organism scientists and the clinicians” (Freeman and St Johnston, 2008). Research published in DMM covers a broad range of scientific topics and model systems. “One of the strengths of DMM is its breadth of model organism coverage – studies using yeast, worm, slime mould, fly, fish, mouse, rat, rabbit, dog, pig and sheep as models have been published”, said Monica Justice (University of Toronto and SickKids Hospital), former Editor-in-Chief (EiC) of DMM, as she likened DMM to a tree with the various model organisms as branches (Justice, 2021). Since then, DMM's scope has expanded to include systems well beyond model organisms. The journal now welcomes research that utilises any system that best advances our understanding of disease mechanism, diagnosis and therapy, including stem-cell-based models, organoids, patient material, model organisms, microorganisms, viruses, computational approaches and other systems with relevance to human disease research. DMM also plays a pivotal role in advancing standards for reproducibility in the biomedical sciences, promoting accurate ways of data reporting and validating model organisms to effectively recapitulate human diseases, while embracing the concept of ‘negative findings’ as an important step towards unabridged science (Siegel, 2011; Matosin et al., 2014; Justice and Dhillon, 2016; Hackett, 2016; Sansom et al., 2024; Juguilon and Wu, 2024; Justice, 2024). In this Editorial, we share a bird's-eye view of DMM's journey since its founding – its growth and maturation from infancy to its current form, under the leadership of four successive EiCs. We also address the journal's commitment to supporting young researchers and promoting research accessibility, along with its recent initiatives in patient advocacy. The accompanying poster illustrates a visual timeline of DMM's 17-year journey; we hope you enjoy taking this ride with us.

## DMM ushers in a new era in disease biology research

In the 2000s, the research community witnessed an increasing focus on disease modelling in experimentally tractable organisms, with conference sessions on disease models appearing particularly “crowded and lively” (Freeman and St Johnston, 2008). Around 2005, Matthew Freeman (Dunn School of Pathology at the University of Oxford) and Daniel St Johnston (Gurdon Institute, University of Cambridge), both Directors at the Company at the time, realised that this rapidly evolving field might remain niche without a dedicated journal. Together, they proposed that the Company investigate the launch of a journal − which would be named Disease Models & Mechanisms (DMM) – that focuses on the use of model organisms to study diseases. Soon after that, the Company's Board of Directors unanimously voted for the launch of DMM as a print and online subscription journal, with Vivian Siegel (Vanderbilt University School of Medicine) as the founding professional EiC. The founders envisioned the new publication to foster dialogue between two scientific cultures that they considered to be poorly connected − model organism scientists and clinicians.

The inaugural issue of DMM was published in July 2008, featuring editorial content and reviews. The cover image of this issue ‘Adam Emergent’, inspired by Michelangelo's Creation of Man, was created by Nadia Rosenthal (The Jackson Laboratory). In the image, Adam is shown to be rising from a bed of model organisms representing the life sciences. In the first Editorial, titled ‘Wherefore DMM?’, Freeman and Johnston wrote, “…we hope that the journal will help medics to encourage the development of new or better models for diseases. This dialogue is not necessarily easy, and may be a slow process, but this taster issue gives a flavour of the ways in which the two cultures can be bridged” (Freeman and St Johnston, 2008). Committed and driven, Vivian Siegel believed that DMM could ‘provoke progress’ in the research community. Beyond conventional review-type front section articles, DMM also started publishing unique sections, such as the ‘A Model for Life’ interview series that showcases leaders in the disease biology community, with the hope of inspiring our then-new readership (Siegel, 2008). Initially, every Research Article published in DMM was accompanied by a Translational Impact piece, which aimed to more simply explain the implications of the research for the clinic (Siegel, 2011). This continued until 2015. However, we now request authors to clearly state the translational impact of a study in their abstract in a way that is accessible to the wider public because we believe that disease impact should be an intrinsic part of a Research Article, rather than a separate piece.

## From an infant to a 17-year-old: DMM's evolution

At DMM, we have been committed to actively supporting the next generation of biomedical scientists since the beginning. The Company's Travelling Fellowships programme was extended to DMM at its launch, enabling early-career researchers (ECRs) to make collaborative visits to other laboratories. With the first issue of DMM, the Company also launched Research Presentation Grants, so that first authors of papers published in DMM could present their work at major scientific conferences. In 2016, this programme evolved into Conference Travel Grants and was extended to all ECRs based on an application process, with the intention of supporting a wider cohort of ECRs, rather than only those who published their work in DMM. Subsequently, we launched an outstanding paper prize, which allows DMM to award prizes every year to the junior author(s) of the paper that is chosen by the journal's Editors to be the most outstanding publication (Hackett, 2018). Recently, we have expanded this award to include a separate prize for Resources & Methods Articles, with the aim of promoting global accessibility to methods and resources pertaining to human disease research (Hooper and Hmeljak, 2022).

With the emergence of social media as an essential part of the publishing landscape, DMM entered the scene in 2010 and is now active on Facebook, X (formerly known as Twitter), Mastodon and Bluesky. We took an important step towards increasing research accessibility when DMM became the Company's first Gold OA journal in 2011. Sharing this news in an Editorial, Vivian Siegel wrote, “All articles will be freely accessible immediately upon publication, distributed under a Creative Commons license and deposited in an open access database” (Siegel, 2011). Additionally, DMM started offering article processing charge (APC) waivers and discounts for authors from low-income and lower-middle-income economies to ensure equity in authors’ ability to publish in DMM.

In the 2010s, the Company's Board of Directors implemented a new editorial model for DMM, shifting from a professional EiC to a team of research-active Editors embedded in their communities, with Vivian Siegel stepping down from her role as EiC in 2013. She compared her experience of launching DMM to that of raising a child and the act of passing the journal over to the new Editors as being at one's child's wedding (Siegel, 2013). Ross Cagan (University of Glasgow) became DMM's EiC, with Senior Editors Monica Justice and George Tidmarsh (La Jolla Pharmaceutical Company and Stanford University School of Medicine) also appointed, together with a team of ‘handling’ Editors. This team was established with the intention that the EiC role would rotate (Hackett, 2016). “My guiding vision is simple: I want to work with my partners to create a merged academic and pharmaceutical community”, Cagan explained in an interview with DMM at the start of his tenure (Cagan, 2013).

The then-new Editors strove to ensure that high standards for translational impact were consistently maintained, while enforcing fair and transparent editorial practices (Hackett, 2016). They aimed to make DMM the ‘go-to’ journal for research using disease models. Soon, their vision started becoming a reality as the number of submissions to DMM doubled, leading to a shift to monthly publication from the previous bimonthly structure. Gradually, special subject collections covering diverse disease areas and the translational impact of different model organisms were introduced (Hackett, 2016). With the emergence of zebrafish as a prominent model in biomedical research, DMM published its first Special Issue, ‘Spotlight on Zebrafish: Translational Impact’, in 2014 with Guest Editors Liz Patton (University of Edinburgh), James Amatruda (University of Southern California) and Lalita Ramakrishnan (MRC Laboratory of Molecular Biology) (Patton et al., 2014).

In alignment with DMM's editorial model, Monica Justice rotated into the EiC position in 2016 and Ross Cagan moved to the Senior Editor role. Around the same time, DMM adopted two-way integration with the bioRxiv preprint server, so authors submitting to DMM could simultaneously deposit their work in bioRxiv and vice versa (Justice, 2021). As an initiative to promote ECRs alongside their work, we launched the ‘First Person’ interview series with the first authors of selected DMM papers (Hackett, 2018). In 2018, DMM moved from issue-based article publication to continuous publication, improving speed to publication of the version of record. We also began offering format-free submission to make manuscript submission as easy as possible for authors. As a part of this change, the Materials and Methods section was excluded from the length limit of an article, enabling authors to describe their methods in sufficient detail for readers to “fully understand and replicate the experiments conducted” (Hackett, 2018). DMM adopted a more collaborative form of peer review by introducing a cross-referee-commenting step that allows our reviewers to comment on the other referee reports before a decision is made, facilitating fair and constructive peer review. All these developments aim to support DMM's community of researchers − be they readers, reviewers or authors.

In 2021, Liz Patton, highly regarded in the zebrafish and DMM communities, succeeded Monica Justice in the role of EiC, following a rigorous recruitment process (Vyas, 2020; Justice, 2021). “We aspire for DMM to become a natural home for science that pushes the boundaries of disease biology and makes strong links with clinical science”, Patton shared in her introductory Editorial, emphasising that quality disease research and accessibility are the twin pillars underpinning the ethos of DMM (Patton, 2021). She wrote, “We believe that the results of scientific research should be accessible to all, including other researchers, clinicians, patients and their families and advocates, their funders and the wider public” (Hackett and Patton, 2022). In line with this commitment to enhancing research accessibility, DMM is now included in the cost-neutral Read & Publish agreements offered by The Company of Biologists, allowing fee-free publication of unlimited Research Articles and Resources & Methods Articles in DMM for corresponding authors at participating institutions. DMM is also an affiliate journal of Review Commons, the journal-agnostic peer review service that facilitates quality peer review. Once reviewed, authors can then submit the manuscript, its peer review reports and their own response to DMM, with the aim of speeding up peer review and reducing reviewer fatigue (Patton, 2024).

At DMM, we believe that patient perspectives are essential for gaining holistic understanding of a disease. To this end, DMM and its sister journal Development organised a virtual meeting on developmental disorders, where patients shared their journeys (Hooper et al., 2021). The patient's voice is important to drive research progress and for improving outcomes for patients (Dorr Goold and Lipkin, 1999). Therefore, in the spirit of paving the most effective way of encouraging disease researchers and clinicians to engage with patients, DMM launched the ‘Building Advocacy into Research’ interview series in 2023, which features interview articles with patients and advocates across various diseases (Amatruda, 2023; Hooper et al., 2024). After listening to patients about their wants and unmet needs, our latest special issue − dedicated to translating research in rare disease − includes a short lay summary accompanying every research-type article to engage and inform patients, their families and advocates, and the general public.

This year marks the 100th birthday of The Company of Biologists (Bray et al., 2025). As a celebratory landmark, we are hosting the ‘Biologists @ 100’ conference at ACC Liverpool, UK from 24-27 March 2025. In addition to combining the spring meetings of the British Society for Cell Biology (BSCB) and the British Society for Developmental Biology (BSDB), this conference will also include the one-day disease-themed programme ‘Interdisciplinary approaches to combatting antimicrobial resistance’ that will be of particular interest to DMM's audience. We hope to see you there.

## ‘Wherefore DMM?’

In the competitive industry of scientific publishing, DMM was launched at a challenging time amid limited library budgets and mounting campaigns demanding improved access to research; however, we thrived and emerged as a one-of-a-kind journal in biomedical research. DMM was, as Ross Cagan put it, “arguably the first journal devoted to merging basic and clinical research, and it has established itself as a flagship journal in translational medicine” (Cagan et al., 2013). We believe in publishing high-quality peer-reviewed research with direct translational impact that goes beyond the traditional metrics of impact and usage. High-quality publications require a high-quality peer review system. We are proud of our peer review at DMM and grateful to those who review for us. To demonstrate this, we publish the names of all our peer reviewers from across the world every year (Patton, 2024). In 2023, The Company of Biologists launched a new biodiversity initiative, called The Forest of Biologists, with support from the Woodland Trust. Through this initiative, we are planting a tree in a UK forest for every Review or Research Article that we publish in DMM and its sister journals. We are also funding the restoration and preservation of ancient woodland, and dedicating these woodland trees to our peer reviewers, who help us to preserve the integrity of the scientific record. You can learn more about this biopositive initiative in Moulton and Freeman (2023).

Together, our Editors and expert members of the Editorial Advisory Board are invaluable in helping DMM publish research and cutting-edge techniques while promoting best publishing practices (Mikimoto and Hooper, 2024). Additionally, our Cambridge-based dedicated in-house team support our Editors, readers, reviewers and authors (Box 1). We thank them all for their hard work, dedication and expertise.

**Box 1. DMM's current in-house team**

The Cambridge-based dedicated in-house team is overseen by our Managing Editor Rachel Hackett, who works closely with DMM's EiC Liz Patton to implement key strategic decisions. Our Features & Reviews Editor Kirsty Hooper and Reviews Editor Dina Mikimoto commission front-section content (i.e. review-type articles) with support from Saanjbati Adhikari (Cross-title Features Editor) and Katie Pickup (Cross-title Reviews Editor). A team of three Production Editors, Akshari Gupta, Claudia Lange and Melissa Ray, meticulously copyedit each issue, correct proofs and ensure proper indexing and archiving. Finally, our Administrators Sue Chamberlain and Debbie Thorpe support Editors, authors, and reviewers throughout the crucial steps of manuscript submission and peer review.

In addition, the Production department carries out quality control checks on published articles, and provides technical support in maintaining our systems. The journal is also supported a research integrity team, who ensure the integrity of the scientific record. The team thanks our colleagues – past and present – for their contribution.

Looking back over the 17 years since DMM's launch, it is striking how much has changed and, no doubt, will continue to change. DMM is lucky to be published by and supported by such a special publisher, a not-for-profit organisation run by scientists for scientists. The Directors of The Company of Biologists (who receive no remuneration) are innovative and supportive, and can be relied on to make decisions for the good of science and scientists. We wish The Company of Biologists a very happy 100th birthday and look forward with excitement to the next 100 years.

## The Company of Biologists: celebrating 100 years

This article is part of ‘The Company of Biologists: celebrating 100 years’ anniversary collection. To view the full collection of articles, please visit: https://journals.biologists.com/journals/pages/celebrating_100_years and, for details of more of our activities happening during 2025, please go to: https://www.biologists.com/100-years/.

## Supplementary Material

10.1242/dmm.052259_sup1 Supplementary information

10.1242/dmm.052259_sup2 Poster
