# Supplementary material for: Travelling through time with Disease Models & Mechanisms
Source: Dis Model Mech. 2025 Feb 24;18(2):DMM052259. doi: 10.1242/dmm.052259 (PMC11904317; doi:10.1242/dmm.052259)
Supplement: Supplementary information [file dmm-18-052259-s1.pdf]

Reviewers for Disease Models & Mechanisms 2024

To ensure that DMM publishes rigorously conducted high-quality research, we are dependent on the time and expertise of our peer reviewers. We take this opportunity to thank all the reviewers and co-reviewers (and reviewers of articles transferred to DMM from Review Commons) who dedicated their time to contributing thoughtful feedback that advances science and how it is communicated.

Pilar Acedo Nunez, University College London, UK

Brian Ackley, University of Kansas, USA

Shaad Ahmad, Indiana State University, USA

Omar Albagha, Hamad Bin Khalifa University, Qatar

Matthew Alexander, University of Alabama at Birmingham, USA

Fowzan Alkuraya, King Faisal Specialist Hospital and Research Center, Saudi Arabia

James Allan, Newcastle University, UK

Lee-Ann Allen, University of Missouri School of Medicine, USA

Martine Ammassari-Teule, Consiglio Nazionale delle Ricerche, Italy

Corina Anastasaki, Washington University in St. Louis, USA

Robin Anderson, Olivia Newton-John Cancer Research Institute, Australia

Eran Andrechek, Michagan State University, USA

Neal Anthwal, King’s College London, UK

Francesco Argenton, Università degli Studi di Padova, Italy

Ruth Arkell, Australian National University, Australia

Jason Arnold, Duke University School of Medicine, USA

Ngan Pan Bennett Au, University of Portsmouth, UK

José Badano, Institut Pasteur de Montevideo, Uruguay

Ana Banito, Deutsches Krebsforschungszentrum, Germany

Mirella Barboni, Semmelweis University, Hungary

Pamela Barraza-Flores, Boston Children’s Hospital, USA

Peter Barr-Gillespie, Oregon Health and Science University, USA

Sina Bartfeld, Technische Universität Berlin, Germany

Inès Barthélémy, Institut national de la santé et de la recherche médicale, France

Caroline Bartman, University of Pennsylvania, USA

Enrico Baruffini, Università degli Studi di Parma, Italy

Victoria Baxter, Texas Biomedical Research Institute, USA

Bilal Bayazit, Nationwide Children’s Hospital, USA

Daniel Becker, University of Oklahoma, USA

Paola Bellosta, University of Trento, Italy

Anat Ben-Zvi, Ben-Gurion University of the Negev, Israel

Sadie Bergeron, West Virginia University, USA

Jason Berman, University of Ottawa, Canada

Jelena Bezbradica, University of Oxford, UK

Thomas Bird, CRUK Scotland Institute, UK

Gregory J. Bix, Tulane University, UK

Kelly Blacklock, The University of Edinburgh, UK

Karen Blyth, CRUK Scotland Institute, UK

Teresa Bonello, Australian National University, Australia

Eliette Bonnefoy, Institut Cochin, France

Dave Boucher, University of York, UK

Luke Boulter, The University of Edinburgh, UK

Sevda Boyanova, University College London, UK

Tomas Brdicka, Czech Academy of Sciences, Czech Republic

Luke Brewster, Emory University, USA

Volker Briken, University of Maryland, USA

Hannah Brunsdon, The University of Edinburgh, UK

Lori Buhlman, Midwestern University, USA

Margret Bülow, Heinrich-Heine-Universität Düsseldorf, Germany

Carol Bult, The Jackson Laboratory, USA

Marie Burdine, University of Arkansas for Medical Sciences, USA

Alexa Burger, University of Colorado, USA

Pilar Cacheiro, Queen Mary University of London, UK

Lindsay Cahill, Memorial University of Newfoundland, Canada

Brian Calvi, Indiana University Bloomington, USA

Francesco Cambuli, New York Genome Center, USA

Nathaniel Campbell, Northwestern University, USA

Cathrin Canto, Netherlands Institute for Neuroscience, The Netherlands

Maria Capovilla, Institut de Pharmacologie Moléculaire et Cellulaire, France

Mathias Carl, University of Trento, Italy

Leo Carlin, CRUK Scotland Institute, UK

Mark Carlson, University of Nebraska Medical Center, USA

Carlos Carmona-Fontaine, New York University, USA

Manuella Cervelli, Università degli Studi Roma Tre, Italy

Matilde Cescon, Università degli Studi di Padova, Italy

Felix Chan, University of Birmingham, UK

Vijayendran Chandran, University of Florida, USA

Bill Chaudhry, Newcastle University, UK

James Chelliah, University of Exeter, UK

Guang-Chao Chen, Academia Sinica, Taiwan

Jing Chen, China Three Gorges University, China

Adam Chicco, Colorado State University, USA

Jessica Chong, University of Washington, USA

Ling-Shiang Chuang, Icahn School of Medicine at Mount Sinai, USA

Martyn Cobourne, King's College London, UK

Justin Cohen, Yale University, USA

Disease Models & Mechanisms • Supplementary information

Rebecca Coll, Queens University Belfast, UK

Ross Collery, Medical College of Wisconsin, USA

Matthieu Colpaert, University of Florida, USA

Christopher Colwell, University of California, Los Angeles, USA

Benjamin Combs, Michigan State University, USA

Fabio Cominelli, Case Western Reserve University, USA

Rob Coppes, Rijksuniversiteit Groningen, The Netherlands

Julia Cordero, University of Glasgow, UK

Silvia Corrochano, Instituto de Investigación Sanitaria del Hospital Clinico San Carlos, Spain

Andrew Cox, Peter MacCallum Cancer Centre, Australia

Marta Cozzi, Università degli Studi di Milano, Italy

Sergio Crespo-Garcia, Université de Montréal, Canada

Richard Cripps, San Diego State University, USA

Mark Cronan, Max-Planck-Institut für Infektionsbiologie, Germany

Gage Crump, University of Southern California, USA

Milena Damulewicz, Jagiellonian University, Poland

Ben Davies, The Francis Crick Institute, UK

Annamaria De Luca, Universita degli Studi di Bari Aldo Moro, Italy

Boel De Paepe, Universiteit Gent, Belgium

Kellie Dean, University College Cork, Ireland

Julien Debbache, Universität Zürich, Switzerland

Cornelia Deeg, Ludwig-Maximilians-Universität München, Germany

Michael Deel, Duke University, USA

Fabio Demontis, St. Jude Children's Research Hospital, USA

Hansong Deng, Tongji University, China

Qing Deng, Perdue University, USA

Jamie DeWitt, East Carolina University, USA

Francesca Di Cara, Dalhousie University, Canada

Amanda Dickinson, Virginia Commonwealth University, USA

Mark Dilworth, The University of Manchester, UK

Albena Dinkova-Kostova, University of Dundee, UK

Martin Distel, St. Anna Children's Cancer Research Institute, Austria

Hakim Djaballah, Keren Therapeutics, USA

Jan Philipp Dobert, Friedrich-Alexander-Universität Erlangen-Nürnberg, Germany

Aloysius Domingo, Massachusetts General Hospital, USA

Zhi-Qiang Dong, The First Affiliated Hospital of Baotou Medical College, China

James Dowling, The Hospital for Sick Children, Canada

Constance Dubois, Centre de recherche du Centre hospitalier de l'Université de Montréal, Canada

Robert Duronio, UNC School of Medicine, USA

Sebastian Dworkin, La Trobe University, Australia

Kevin Dzobo, University of Cape Town, South Africa

Oliver Eichmueller, Österreichischen Akademie der Wissenschaften, Austria

Stephanie Eid, University of Michigan, USA

Marc Ekker, University of Ottawa, Canada

Elie El Agha, Justus-Liebig-Universität Gießen, Germany

Philip Elks, University of Sheffield, UK

Lisa Ellerby, Buck Institute for Research on Aging, USA

Maria Ermolaeva, Leibniz Institute on Aging, Germany

Todd Evans, Weill Cornell Medicine, USA

Kimberley Evason, Huntsman Cancer Institute, USA

Steven Farber, Johns Hopkins University, USA

Hui Feng, Boston University, USA

Beata Filipek-Gorniok, Uppsala Universitet, Sweden

Rita Fior, Champalimaud Foundation, Portugal

Anthony Firulli, Indiana University School of Medicine, USA

Oliver Fischer, Bayer, Germany

Elizabeth Fisher, University College London, UK

Tatiana Flisikowska, Technische Universität München, Germany

Marycruz Flores Flores, University of Colarado, USA

Xiao Fu, CRUK Scotland Institute, UK

Takahisa Furukawa, Osaka University, Japan

Irene Gallego Romero, St Vincent's Institute of Medical Research, Australia

Subramaniam Ganesh, Indian Institute of Technology, India

Joan Garrett, University of Cincinnati, USA

Zev Gartner, University of California San Francisco, USA

Melanie Gartz, Medical College of Wisconsin, USA

Kinga Gawel, Medical University of Lublin, Poland

Matthew Gentry, University of Florida, USA

Mehrnaz Gharaee-Kermani, University of Michigan, USA

Piya Ghose, The University of Texas at Arlington, USA

Ed Giniger, National Institute of Neurological Disorders and Stroke, USA

Maurizio Giustetto, Università degli Studi di Torino, Italy

Leanne Godinho, Technische Universität München, Germany

Annie Godwin, University of Portsmouth, UK

Wolfram Goessling, Harvard Medical School, USA

Rocco Gogliotti, Loyola University Chicago, USA

Yogesh Goyal, Northwestern University, USA

Todd Graham, Vanderbilt University, USA

Stephanie Grainger, Van Andel Institute, USA

Nicholas Greene, University College London, UK

Disease Models & Mechanisms • Supplementary information

Agi Grigoriadis, King's College London, USA

Stephane Gross, Aston University, UK

Miranda Grounds, The University of Western Australia, Australia

Markus Gugatschka, Medical University of Graz, Austria

Vandana Gupta, Brigham and Women's Hospital, USA

Asiya Gusa, Duke University School of Medicine, USA

Mohammad Hajihosseini, University of East Anglia, UK

Alex Hajnal, Universität Zürich, Switzerland

Bengt Hallberg, Göteborgs Universitet, Sweden

Ada Hamosh, Johns Hopkins University, USA

Zhe Han, University of Maryland School of Medicine, USA

Natasha Hanners, UT Southwestern Medical Center, USA

Hanaa Hariri, Wayne State University, USA

Matthew Harris, Harvard Medical School, USA

Patrick Harrison, University College Cork, Ireland

Okado Haruo, Tokyo Metropolitan Institute of Medical Science, Japan

Xue-Yan He, Washington University School of Medicine, USA

Christopher Heier, Virginia Commonwealth University, USA

Ilka Heinemann, Western University, Canada

Michael Heinrich, Oregon Health and Science University, USA

Filipa Henderson Sousa, The University of Edinburgh, UK

Tobias Hermle, Universität Freiburg, Germany

Gary Hime, The University of Melbourne, Australia

Catherine Hogan, Cardiff University, UK

Marshall Hogarth, Children's National Research Institute, USA

Stephen Holland, CHEO Research Institute, Canada

Bo Hu, Army Medical University, China

Huili Hu, Shandong University, China

Kang-Cheih Huang, Baylor College of Medicine, USA

Shushu Huang, Yale University, USA

Xupei Huang, Florida Atlantic University, USA

Sabah Hussain, McGill University, Canada

Tatsushi Igaki, Kyoto University, Japan

Akihiro Ikeda, University of Wisconsin–Madison, USA

Pascal Imbeault, University of Ottawa, Canada

Tohru Ishitani, Osaka University, Japan

Yosuke Ishitsuka, Osaka University, Japan

Anthony Isles, Cardiff University, UK

Evgueni Ivakine, The Hospital for Sick Children, Canada

Daniyal Jafree, University College London, UK

Pudur Jagadeeswaran, University of North Texas, USA

Sanjay Jain, Johns Hopkins Medicine, USA

Marnix Jansen, University College London, UK

Kimberly Jasmer, University of Missouri, USA

Keaton Ian Jones, University of Oxford, UK

Monica Justice, The Hospital for Sick Children, Canada

Vesa Kaartinen, University of Michigan, USA

Erika Kague, The University of Edinburgh, UK

Michael Kahn, Beckman Research Institute of City of Hope, USA

Daichi Kamiyama, University of Georgia, USA

Fuyuki Karube, Hokkaido University, Japan

Ajith Karunarathne, Saint Louis University, USA

Yoshitaka Kawai, Kyoto University, Japan

Genevieve Kendall, Nationwide Children's Hospital/The Ohio State University, USA

Emma Kerr, Queen's University Belfast, UK

Benard Khor, Benaroya Research Institute, USA

Un-Kyung Kim, Kyungpook National University, Republic of Korea

Junzo Kinoshita, Daiichi Sankyo, Japan

Stefanie Kirchberger, St. Anna Children's Cancer Research Institute, Austria

Alfredo Kirkwood, Johns Hopkins University, USA

David Kirsch, University of Toronto, Canada

Janine Kirstein, Leibniz Institute on Aging, Germany

Sybille Köhler, Universitätsklinikum Hamburg-Eppendorf, Germany

Yoshihiro Komatsu, The University of Texas Health Science Center at Houston, USA

Jeffrey Kopp, National Institute of Diabetes and Digestive and Kidney Diseases, USA

Doris Kretzschmar, Oregon Health and Science University, USA

Warren Kruger, Fox Chase Cancer Center, USA

Michael Kühl, Universität Ulm, Germany

Ashwani Kumar, Yale University, USA

Driederik Kuster, Amsterdam UMC, The Netherlands

Yun Kwon, Medizinische Fakultät Mannheim, Universität Heidelberg, Germany

Maris Laan, University of Tartu, Estonia

Matthias Lambert, Boston Children's Hospital, USA

Karen Lange, University College Dublin, Ireland

Francesca Lavatelli, Università degli Studi di Pavia, Italy

Anna-Lisa Lawrence, Tufts University School of Medicine, USA

Robert Layfield, University of Nottingham Medical School, UK

Monkol Lek, Yale University, USA

Rachel Lennon, The University of Manchester, UK

Jack Leslie, Newcastle University, UK

Yuk Fai Leung, Purdue University, USA

Disease Models & Mechanisms • Supplementary information

Thomas Liehr, Universitätsklinikum Jena, Germany

Hui Lim, University of Oklahoma Health Sciences Center, USA

Angus Lindsay, University of Canterbury, New Zealand

Qinglan Ling, University of Massachusetts Chan Medical School, USA

Karen Liu, King's College London, UK

Hanns Lochmüller, CHEO Research Institute, Canada

Katie Long, King's College London, UK

Hannah Long, The University of Edinburgh, UK

Jose Lopez-Escamez, The University of Sydney, Australia

Yonglun Luo, Aarhus University, Denmark

Gordon Lynch, The University of Melbourne, Australia

David Lyons, The University of Edinburgh, UK

Kaiyue Ma, Shanghai Jiao Tong University, China

Qinxi Ma, University of Exeter, UK

Samuel Mackenzie, University of Rochester Medical Center, USA

Thomas MacVicar, University of Glasgow, UK

Iddo Magen, Weizmann Institute of Science, Israel

Geoffrey Maher, Imperial College London, UK

Gretel Major, University of Canterbury, New Zealand

Tomoko Makishima, University of Texas Medical Branch, USA

Vivek Malhotra, Centre de Regulació Genòmica, Spain

Zoe Mann, King's College London, UK

M. Chiara Manzini, Rutgers University, USA

Maria Marchese, IRCCS Fondazione Stella Maris, Italy

John Mariadason, Olivia Newton-John Cancer Research Institute, Australia

Joseph Marsh, The University of Edinburgh, UK

Alfonso Martín-Pena, University of Florida, USA

Jean-Francis Mauger, University of Ottawa, Canada

Michelle Maugham-Macan, University of the Sunshine Coast, Australia

Heather McCauley, University of North Carolina at Chapel Hill, USA

Barry McColl, The University of Edinburgh, UK

Sue McGlashan, The University of Auckland, New Zealand

Serge McGraw, Université de Montréal, Canada

Gerry McLachlan, The University of Edinburgh, UK

Roly Megaw, The University of Edinburgh, UK

Annemarie Meijer, Universiteit Leiden, The Netherlands

Luis Fernando Menezes, National Institute of Diabetes and Digestive and Kidney Diseases, USA

Madhav Menon, Yale University, USA

Diane Merry, Thomas Jefferson University, USA

Mohamad Mikati, Duke University, USA

Richard Mills, Murdoch Children’s Research Institute, Australia

Yuji Mishina, University of Michigan, USA

Richra Mishra, Universität Heidelberg, Germany

Florence Molinari, Marseille Medical Genetics, France

Mervyn Monteiro, University of Maryland, USA

Sally Moody, George Washington University, USA

Bethany Moore, University of Michigan, USA

Jennifer Morton, CRUK Scotland Institute, UK

Christian Mosimann, University of Colorado, USA

Darrell David Mousseau, University of Saskatchewan, Canada

Daniel Murphy, University of Glasgow, UK

Aaron Nagiel, Children's Hospital Los Angeles/University of Southern California, USA

Teresa Niccoli, University College London, UK

Sophie Nicole, Institut national de la santé et de la recherche médicale, France

David Nikolic-Paterson, Monash University, Australia

Elke Ober, Friedrich-Alexander-Universität Erlangen-Nürnberg, Germany

Rainer Oberbauer, Medical University of Vienna, Austria

Lori O'Brien, University of North Carolina at Chapel Hill, USA

Karen Ocorr, Sanford Burnham Prebys Medical Discovery Institute, USA

Stefan Oehlers, Agency for Science Technology and Research, Singapore

Ali Oghabian, University of Helsinki, Finland

Mikiko Oka, Baylor College of Medicine, USA

Kiel Ormerod, Middle Tennessee State University, USA

Edward Owusu-Ansah, Columbia University, USA

Antonio Pagán, Stanford University School of Medicine, USA

Michael Palladino, University of Pittsburgh, USA

Daniela Panakova, Universitätsklinikum Schleswig-Holstein, Campus Kiel, Germany

Angela Panoskaltsis-Mortari, University of Minnesota, USA

Hae-Ryung Park, University of Rochester, USA

Jong Hoon Park, Sookmyung Women's University, Republic of Korea

Alex Parker, Centre de recherche du Centre hospitalier de l'Université de Montréal, Canada

William Parker, PNP Therapeutics, USA

Michelle Parvatiyar, Florida State University, USA

Christopher Pastras, Macquarie University, Australia

Manav Pathania, Cambridge University, UK

Liz Patton, The University of Edinburgh, UK

Jinrong Peng, Zhejiang University, China

Weng Chuan Peng, Princess Máxima Center for Pediatric Oncology, The Netherlands

Danielle Peterse, Boston Children's Hospital, USA

Capucine Picard, Hôpital Universitaire Necker, France

Disease Models & Mechanisms • Supplementary information

Richard Piercy, Royal Veterinary College, UK

Stefan Pinter, University of Connecticut, USA

Angelo Poletti, University of Milan, Italy

Rituraj Purohit, CSIR - Institute of Himalayan Bioresource Technology, India

Sonja Pyott, Rijksuniversiteit Groningen, The Netherlands

Mattia Quattrocelli, Cincinnati Children's Hospital Medical Center, USA

Suresh Rattan, Aarhus University, Denmark

David Reiner, Texas A&M University, USA

Stephen Renshaw, University of Sheffield, UK

David Rice, University of Helsinki, Finland

Jesus Rivera-Nieves, University of California San Diego, USA

Ed Roberts, CRUK Scotland Institute, UK

Paul Robinson, University of Oxford, UK

Christian Rocheleau, Research Institute of the McGill University Health Centre, Canada

Daniel Rodrigo Torres, The University of Edinburgh, UK

Emily Rosowski, Clemson University, USA

Frederick Roth, University of Pittsburgh, USA

Jeffrey Rothstein, Johns Hopkins Medicine, USA

Loris Russo, Università degli Studi di Padova, Italy

Anguraj Sadanandam, The Institute of Cancer Research, UK

Sakthivel Sadayappan, The University of Arizona, USA

Stephan Safe, Texas A&M University, USA

Marina Saito, University of Texas Medical Branch, USA

Kei Sakamoto, University of Copenhagen, Denmark

Mootaz Salman, University of Oxford, UK

Lisa Sandell, University of Louisville, USA

Vijay Sankaran, Boston Children's Hospital, USA

Filippo Santorelli, IRCCS Fondazione Stella Maris, Italy

Smita Saxena, University of Missouri, USA

John Sayer, Newcastle University, UK

Elenora Scalia, The University of Edinburgh, UK

Angelika Schnieke, Technische Universität München, Germany

Mark Schroeder, Washington University in Saint Louis, USA

Erin Seeley, University of Texas at Austin, USA

Alan Serrels, The University of Edinburgh, UK

Kunming Shao, Purdue University, USA

Florian Siebzenrubl, Cardiff University, UK

Michael Sigal, Charité – Universitätsmedizin Berlin, Germany

Roy Sillitoe, Baylor College of Medicine, USA

Christophe Sirac, Université de Limoges, France

Joanna Smeeton, Columbia University, USA

Zachary Smith, Yale University, USA

Ian Smyth, Monash University, Australia

Lukas Sommer, Universität Zürich, Switzerland

Hermona Soreq, The Hebrew University of Jerusalem, Israel

Viktoriya Stancheva, University of Oxford, UK

Josefa Steinhauer, Yeshiva University, USA

Rolf Stottmann, Nationwide Children's Hospital, USA

Brian Stramer, King's College London, UK

Stanley Strawbridge, Cambridge Stem Cell Institute, UK

Atsushi Sugie, Niigata University, Japan

Chih Sung, Tri-Service General Hospital, Taiwan

Atsushi Suzuki, Hiroshima University, Japan

Karolina Swiderska, Université de Limoges, France

Kristy Swiderski, The University of Melbourne, Australia

Mazazumi Tada, University College London, UK

Ken Takahashi, Okayama University, Japan

Jared Talbot, The University of Maine, USA

Vikram Tallapragada, Victor Chang Cardiac Research Institute, Australia

David Talmage, National Institute of Neurological Disorders and Stroke, USA

Owen Tamplin, University of Wisconsin–Madison, USA

Shumin Tan, Tufts University School of Medicine, USA

Li Xuan Tan, University of Health and Rehabilitation Sciences, China

Aleksandra Tata, Duke University School of Medicine, USA

Simon Tew, University of Liverpool, UK

Vivek Thacker, Universität Heidelberg, Germany

Arianne Theiss, University of Colorado, USA

Praveen Thumbikat, Northwestern University, USA

Aiguo Tian, Tulane University, USA

Randal Tibbetts, University of Wisconsin–Madison, USA

David Tobin, Duke University, USA

Laszlo Tora, Institute of Genetics and Molecular and Cellular Biology, France

Judit Tóth, HUN-REN Research Centre for Natural Sciences, Hungary

DeWayne Townsend, University of Minnesota, USA

Pamela Tran, University of Kansas Medical Center, USA

Karla Troncoso, University of North Carolina at Chapel Hill, USA

Rodrigo Troncoso Cotal, Universidad de Chile, Chile

Simon Trowitzsch, Goethe-Universität Frankfurt am Main, Germany

Aaron Tward, University of California San Francisco, USA

Steve Twigg, Oxford University, UK

Cyrille Vaillend, Institut des Neurosciences Paris-Saclay, France

Ainara Vallejo, Biogipuzkoa Health Research Institute, Spain

Tom Van Agtmael, University of Glasgow, UK

Ludo Van Den Bosch, KU Leuven, Belgium

Maaike van Putten, Leiden University Medical Center, The Netherlands

Jeremy Van Raamsdonk, McGill University, Canada

Johan Vande Voorde, University of Glasgow, UK

Davy Vanhoutte, Cincinnati Children's Hospital Medical Center, USA

A. Catalina Velez-Ortega, University of Kentucky, USA

Esther Verheyen, Simon Fraser University, Canada

Mayank Verma, UT Southwestern Medical Center, USA

Hilary Vernon, Johns Hopkins Medicine, USA

Tatyana Vetter, Nationwide Children's Hospital, USA

Divya Vimal, Columbia University, USA

Stephane D. Vincent, Institute of Genetics and Molecular and Cellular Biology, France

Benoit Viollet, Institut Cochin, France

Margreet C. M. Vissers, University of Otago, New Zealand

Veronique Vitart, The University of Edinburgh, UK

Cindy Voisine, Northeastern Illinois University, USA

Pierre-Yves von der Weid, University of Calgary, Canada

Alex von Kriegsheim, The University of Edinburgh, UK

Edith Wang, University of Washington, USA

Yi Wang, Boston Children's Hospital, USA

Yi-Ting Wang, Ohio University, USA

Christopher Ward, Baylor College of Medicine, USA

Yuli Watanabe, Institut national de la santé et de la recherche médicale, France

Noah Weisleder, University of Kentucky, USA

Dominic Wells, Royal Veterinary College, UK

Disease Models & Mechanisms • Supplementary information

Theodore Wensel, Baylor College of Medicine, USA

Robert Wheeler, The University of Maine, USA

Alexander Whitworth, University of Cambridge, UK

Ulrich Wilk, Ludwig-Maximilians-Universität München, Germany

Meredith Wilson, Johns Hopkins University, USA

Chengbiao Wu, University of California San Diego, USA

Ranjie Xu, Purdue University, USA

Kazuhiko Yamada, Johns Hopkins Medicine, USA

Shinya Yamamoto, Baylor College of Medicine, USA

Yojiro Yamanaka, McGill University, Canada

Hongyuan Yang, University of New South Wales, Australia

Kai-Chun Yang, University of Washington, USA

Yun You, University of Minnesota, USA

Michael Zech, Technische Universität München, Germany

Anja Zeigerer, Medizinische Fakultät Mannheim, Universität Heidelberg, Germany

Friederike Zunke, Friedrich-Alexander-Universität Erlangen-Nürnberg, Germany
