# Supplementary figures and images for: Travelling through time with Disease Models & Mechanisms
Source: Dis Model Mech. 2025 Feb 24;18(2):DMM052259. doi: 10.1242/dmm.052259 (PMC11904317; doi:10.1242/dmm.052259)

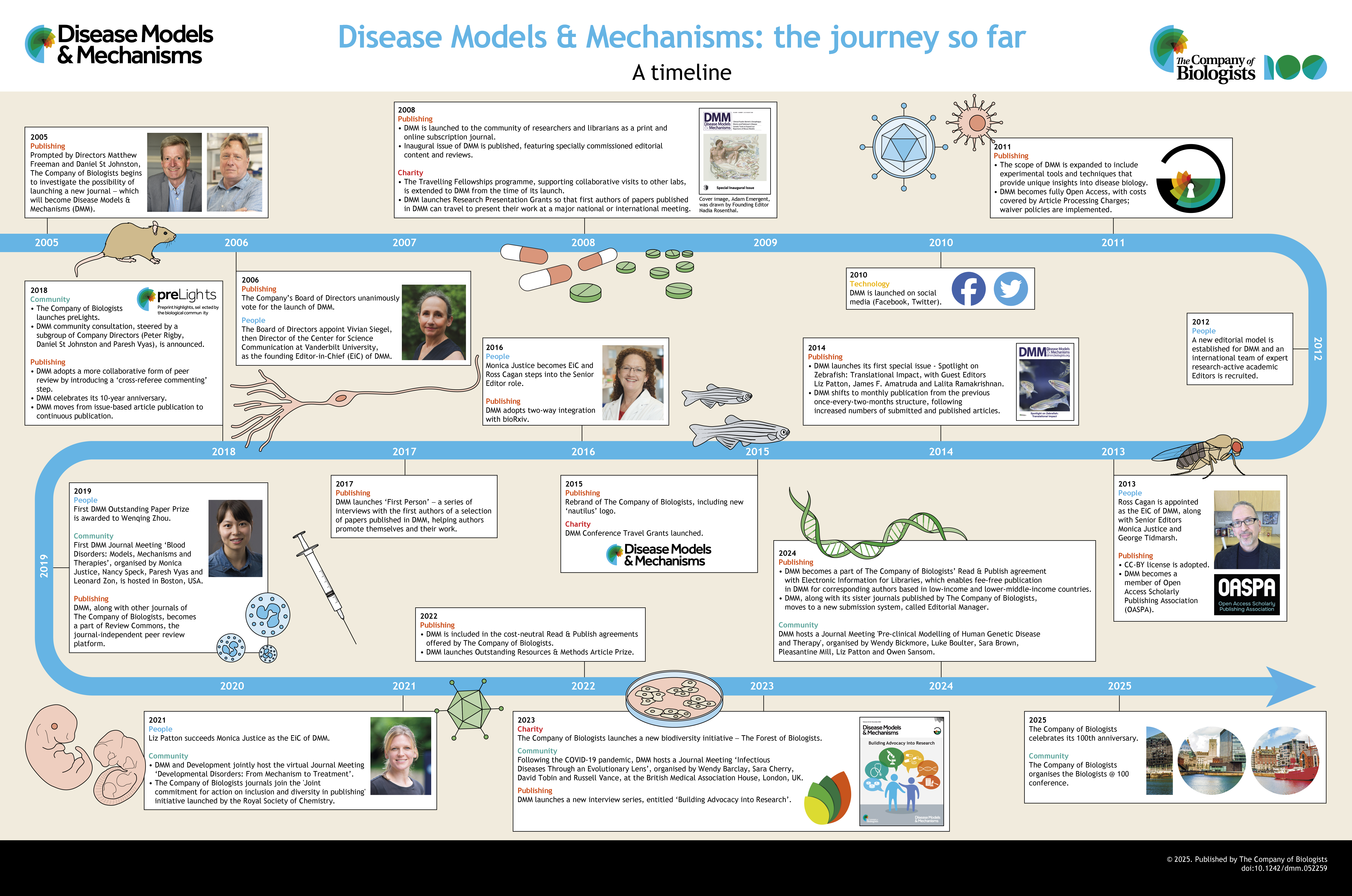

Supplement: Poster [file dmm-18-052259-s1.jpg]
